# Chronic Kidney Disease and Older African American Adults: How Embodiment Influences Self-Management

**DOI:** 10.3390/geriatrics3030052

**Published:** 2018-08-16

**Authors:** Tyrone C. Hamler, Vivian J. Miller, Sonya Petrakovitz

**Affiliations:** 1 Mandel School of Applied Social Sciences, Case Western Reserve University, Cleveland, OH 44106, USA; 2 School of Social Work, University of Texas at Arlington, Arlington, TX 76109, USA; vivian.miller@mavs.uta.edu; 3 Department of Anthropology, Case Western Reserve University, Cleveland, OH 44106, USA; sonya@case.edu

**Keywords:** chronic disease, self-management, health disparities

## Abstract

Patients living with chronic kidney disease (CKD) must balance the medical management of their kidney disease and other chronic conditions with their daily lives, including managing the emotional and psychosocial consequences of living with a chronic disease. Self-management is critical to managing chronic kidney disease, as treatment consists of a complex regimen of medications, dosages, and treatments. This is a particularly important issue for older African American adults who will comprise a significant portion of the older adult population in the coming years. Yet current conceptualizations of self-management behaviors cannot adequately address the needs of this population. Embodiment theory provides a novel perspective that considers how social factors and experiences are embodied within decision-making processes regarding self-management care among older African Americans. This paper will explore how embodiment theory can aid in shifting the conceptualization of self-management from a model of individual choice, to a framework that cannot separate lived experiences of social, political, and racial factors from clinical understandings of self-management behaviors. This shift in the conceptualization of self-management is particularly important to consider for CKD management because the profound illness burdens require significant self-management and care coordination skills.

## 1. Introduction

Patients living with chronic kidney disease must balance the medical management of their kidney disease and other chronic conditions with their daily lives, including managing the emotional and psychosocial consequences of living with a chronic disease [1]. Self-management is critical to managing chronic kidney disease since treatment consists of a complex regimen of medications and treatments. This is a particularly important issue for older African American adults who will comprise a significant portion of the older adult population in the coming years. Current conceptualizations of self-management behaviors cannot adequately address the needs of this population. This paper will explore how embodiment theory can aid in shifting the conceptualization of self-management from a model of individual choice, to a framework that cannot separate lived experiences of social, political, and racial factors from clinical understandings of self-management behaviors. By assuming all patients have complete agency over their disease management, the current model of self-management ignores the very real structural, social, and cultural forces that limit patient options and decisions. When we prioritize the context in which older African American patients must negotiate their care, we are also then removing the moral, judgement-based undertones of their self-management behaviors if they are unable to adhere to idealized treatment targets. This shift in the conceptualization of self-management is particularly important to consider for chronic kidney disease management because the profound care burdens of this illness require significant self-management and care coordination skills.

Between 2016 and 2030, the white (non-Hispanic) population aged 65 and over is projected to increase by 39% compared with 89% for older racial and ethnic minority populations, including African Americans (non-Hispanic; 73%) [2]. As the baby boomer population continues to age and the older adult population becomes more racially diverse than ever before, chronic disease self-management will become increasingly important among older African Americans. This has been recognized nationally through legislative initiatives targeting older adults with chronic illnesses including, most recently, the CHRONIC Care Act of 2017 extending and expanding the Independence at Home demonstration program, through which comprehensive primary care services are delivered at home to Medicare beneficiaries with multiple chronic conditions [3].

### 1.1. Chronic Kidney Disease

Chronic kidney disease (CKD) is an emerging public health problem. Chronic kidney disease is defined according to the presence of kidney damage and level of kidney function for a time period of longer than three months [4]. CKD is staged by severity from 1 to 5, with Stages 4 and 5 being indicative of severely decreased kidney function and End-Stage Renal Disease (ESRD), respectively. Kidneys that function properly are critical for maintaining overall good health; however, one in seven adults in the United States are estimated to have chronic kidney disease [5].

There are multiple health outcome disparities in chronic kidney disease for African Americans. These include disparities in CKD prevalence [6] and CKD progression [7]. In advanced stages of CKD, African Americans over age 65 have death rates 15% greater than Whites in the same age range [8]. African Americans are disproportionately diagnosed with diabetes and hypertension, which are respectively the two leading causes of CKD. Microvascular disease resulting from diabetes can cause CKD in about 40–50% of patients with diabetes, a process called diabetic nephropathy [9]. Hypertension (HTN) and CKD are closely associated; as blood pressure rises, typically kidney function declines, and sustained elevations in blood pressure hasten the progression of kidney disease [10]. There are approximately 30 million people living with CKD and millions of other people potentially at risk for developing CKD [11]. The estimated lifetime risk of developing moderate CKD (Stages 3, 4) is 59% for all adults—considerably higher than the 33–39% lifetime risk of diabetes—and among low-income and minority groups, especially African Americans, the prevalence of CKD is higher with a faster progression to end-stage renal disease [12]. CKD exacts a particular care burden on individuals living with this illness. Patients with CKD had approximately twice as many prescriptions, had 1.3–1.9 times as many office visits, and were 1.6–2.2 times as likely to be hospitalized [13]. Additionally, the symptom burden of CKD has been described as comparable to that of individuals undergoing chemotherapy treatment [14].

Older African Americans will comprise a significant population of older adults entering the renal care system in the years to come. Older adults are already among the fastest growing population entering the renal care system, and this group has a particularly high cost burden on Medicare spending when comparing chronic kidney disease to other Medicare-covered diseases. In 2015, Medicare spending for beneficiaries with CKD aged 65 and older exceeded $55 billion, representing 20% of all Medicare spending in this age group [11]. CKD also exacts a significant cost burden on patients who have yet to progress to ESRD. In a study comparing the costs of medical care for CKD patients to age-matched controls, researchers found overall healthcare costs were $14,000 to $22,000 more per year for CKD patients [15]. The clearest economic impact of CKD is understood when considering the cost to Medicare for individuals who progress to ESRD. This included over $64 billion in spending for all Medicare beneficiaries who have CKD and a further $34 billion for beneficiaries with ESRD [11].

### 1.2. Older African Americans

As a group, older African Americans continue to experience socioeconomic, political, and health consequences of structural racism which impact how they seek and perceive care. For older African Americans, racial identity impacts navigation through the healthcare system, thus impacting chronic illness self-management behaviors. Older African American adults born between approximately 1940 and 1960 grew up in a time where Jim Crow legislation (1880s–1960s) still enforced legalized segregation in the United States. Prior to the reversal of Jim Crow, African Americans were politically excluded from participation and representation in United States society. Jim Crow legislation allowed for legalized racial discrimination at county, city, and state levels in many states. The Jim Crow polity consisted of the District of Columbia and the 21 states whose state laws, prior to enactment of the 1964 U.S. Civil Rights Act, legalized racial discrimination in one or more of the following domains: education, transportation, hospital and penal institutions, welfare institutions (pauper homes), employment, marriage, public accommodation, other public venues, and voting [16]. This impacted several areas of human services including access to, and quality of, healthcare services. Hospitals and other medical institutions were segregated in states that enforced Jim Crow laws [17].

The current population of Black older adults grew up as children or young adults during this time and were directly and indirectly exposed to the impacts of this legislation. Older African Americans who experienced these racist and discriminatory laws ostensibly experienced psychological and emotional distress, likely threatening not only their physical safety but their embodied health as well [18]. These experiences have undoubtedly impacted how African Americans receive care and treat illness. Among this group of African Americans, the maintenance of indigenous traditions of self-care was essential for survival in the South under Jim Crow and in northern cities [19]. The historical context that informs older African Americans’ self-management strategies is integral to understanding how individuals understand, manage, and treat their illnesses. The historical necessities of self-management are an important concept in understanding how older African Americans view and experience chronic illness.

### 1.3. Self-Management

Self-management (SM) has traditionally been defined by medical professionals as the process by which an individual assumes responsibility for their own welfare and behavior [20]. SM researchers argue that patients are in control of their own health, regardless of the health providers’ recommendations and warnings [21]. While this system, on the individual level, encourages patients to assume responsibility for their own health, this approach is nested within a larger medical context of a patient support, or person-centered, model of care delivery [22,23]. This idealized process of self-management includes developing a health management plan with a healthcare provider, using behavioral modification to manage disease symptoms (e.g., exercise or meal planning), and active participation in goal and care planning [20]. In summation, this concept of chronic disease self-management, cited across the seminal work of [23], consists of three primary areas: (1) medical guidance, (2) behavior/role management, and (3) emotional management.

These characteristics of SM are found to be in line with features of successful aging, which include adaptation, maintaining a positive mind, achieving personal competence, and taking initiative through self-motivation [24,25]. Within existing literature [26], SM is effective for users concerning self-efficacy, psychological health status, and health behaviors. In theory, individuals with the help of healthcare practitioners, family, and community members are able to manage disease symptoms, treatments, changes in lifestyle, and any cultural, spiritual, and psychosocial consequences as a result of the persisting health condition [27]. SM is considered especially important and unique for older adults. Schulman et al. [27] cite research [28] which reports that older adults with chronic conditions do not want their children to be burdened with providing care. Although this research suggests that older adults may engage in SM to mitigate the risk of such feelings of burden, and perhaps other emotional consequences, only the successful ability for older adults to engage in self-management behaviors has been associated with an increase in well-being and decreased levels of depression [24]. The goals of these self-management interventions and approaches to personalized care are to improve overall health outcomes in general, and particularly for African-American older adults; this has been the case in some studies [29,30,31,32].

Yet we argue that the current framework of self-management as a model of individual choices and behaviorally based decisions fundamentally ignores the complexities of human experience and the actual ability of patients to “take control” of their chronic illness management. If we add a clinical consideration of embodiment theory to the goals of self-management, a new arrangement of care emerges: an approach that cannot separate a patient’s awareness of their illness narratives, physical disease progression, and lived experiences of social, political, and racial factors from their self-management behaviors. This new approach broadens the scope and applicability of self-management, encouraging clinicians to consider patients as holistic beings instead of a diagnosis and prognosis. The incorporation of embodiment theory further removes the moral judgements placed on patients if they are unable to adhere to the precise regimens needed in chronic kidney disease management. Instead of placing culpability of “non-compliance” fully on the locus of the patient and their presumed inability to hold themselves accountable for their own care, incorporating the anthropological understanding of embodiment theory from the beginning of treatment plan construction reframes “the person as a bad patient” into “the patient in a bad situation”. While we cannot remove the tangible need for some level of personal responsibility within chronic disease management, if we (as a society and as medical professionals) are able to incorporate understandings of the embodied “Other”, then we believe it will lead to more comprehensive and realistic, truly patient-centered care plans. Thus, instead of applying an idealized and universalized treatment plan to patients with inequitable resources, and then blaming them for their inability to meet unachievable goals, we can focus on the context that patients inhabit and how to manage their care within that context.

### 1.4. Older African Americans and Self-Management

Older African Americans are diagnosed with chronic diseases and persistent health conditions at a disproportionately higher rate compared with their white counterparts [33]. General research in self-management and self-care among older African Americans with chronic illnesses has found that this group employs several coping strategies when approaching self-care including medication management, spirituality, and engaging in life [34,35,36]. In one study concerning self-management among older African Americans, community members were provided with a Chronic Disease Self-Management Education Program (CDSMEP). This study suggested that African Americans are diagnosed with chronic conditions earlier in life, thus giving them the sense that they know how to successfully manage their chronic condition and do not need programming to learn to do so [32]. In a qualitative analysis, Philips et al. [32] found that older African Americans developed strong SM *coping skills*, primarily an acceptance and understanding of their condition, reliance on religiosity and spirituality by using faith to cope, and reliance on family and community support. Despite these findings of positive SM behaviors among this group, additional research [32,37,38,39] has found that African Americans tend to have decreased levels of SM behaviors, such as medication management. 

Empirical research focusing specifically on older African Americans and self-management is limited; however, there has been work that has concentrated on how older African Americans manage various chronic conditions. There have been several inquiries concerning diabetes self-management and older African Americans. One such study examined cultural beliefs and adherence to self-management behaviors among a community-based sample (*N* = 100) of older African American adults and found that participants who engaged in three specific behavioral areas—regular exercise, reading food labels, and checking blood glucose—had significantly lower present time orientation scores, higher future time orientation, and religiosity scores; higher future orientation scores have been linked to health-promoting behaviors [40]. Another inquiry focused on employing a culturally targeted diabetes self-management intervention—which included diabetes education, stress management, coping skills, and physical activity for twelve African American middle-aged and older adults in urban churches—found that church-based cultural interventions were effective in reinforcing diabetes education [41]. Several inquiries focused on hypertension self-management. Flynn et al. [39] identified facilitators among African American adults (mean age = 61) to self-management care, such as spirituality, family support, positive provider–patient relationship, and community-based support, and barriers such as comorbidities, poor knowledge of the disease, long clinic wait times, and lack of resources to check Blood Pressure between doctor’s visits. Klymko et al. [42] looked at self-care production among African Americans ages 60–89 living with hypertension and discovered that older adults’ experiences with the production of self-care took on three themes: preparation, monitoring, and evaluation. These tasks were often viewed as necessitating additional social support assisting this group of older adults. Warren-Findlow, Seymour, and Shenk [43] examined whether intergenerational transmission of hypertension knowledge and self-efficacy would affect hypertension self-care of older parents and their adult children, and found that parents are more adherent to medication regimens than their adult children diagnosed with hypertension, and that interventions should target older adults who can serve as influencers within the family. Ibrahim, Siminoff, Burant, and Kwoh [44] found older African Americans with osteoarthritis were likely to be receptive to self-care and view this as efficacious, while older Whites were more likely to view joint replacement as an effective treatment for osteoarthritis. Another inquiry examining osteoarthritis and older African Americans found that African Americans limited their activity or rested much less than Whites throughout the day, yet African Americans used other strategies more often, such as taking medications throughout the day and using topical treatments regularly and at bedtime [45]. Studying older African American women with early stage heart disease has identified social support as an important factor for older women, with instrumental support such as providing transportation and financial help being of particular importance [46].

### 1.5. Chronic Kidney Disease and Self-Management

Chronic kidney disease self-management is an important emerging area of inquiry. Research concerning self-management and older adults is limited, with scant literature specifically focusing on older African Americans. In general, irrespective of age, previous inquiries in chronic kidney disease have found the following variables to be significant regarding self-management: self-efficacy [47,48,49], medication adherence [50,51], and self-care [52]. Additionally, there have been various self-management interventions used within this population, predominantly provided by nurses and dieticians. These interventions include exercise programs [53,54], mindfulness meditation [55], self-management of medication programs [56,57], and dietary self-management interventions [58,59] High levels of self-efficacy and low levels of self-advocacy were reported in a sample (*N* = 174) that was 64% African American with a mean age of 50.9 years [51]. Perceived self-efficacy was associated positively with communication behaviors, partnership behaviors, self-care activities, and medication adherence [51]. In a qualitative study of factors influencing self-management (*N* = 107, 65% African American with a mean age of 63 years), it was found that despite the overall low mean scores in exercise, communication with physicians, and cognitive symptom management, participants reported high self-efficacy over self-management tasks [52]. In an examination of a structured exercise program with obese chronic kidney disease patients diagnosed with diabetes (*N* = 36, *n* = 13 were African American), structured exercise on a treadmill improved physical function, but did not improve eGFR (a standard measure of kidney function) [53]. A randomized crossover study of 15 African American men diagnosed with hypertensive CKD utilizing mindfulness meditation found that mindfulness meditation acutely lowers BP and Heart Rate in African American males, and these hemodynamic effects may be mediated by a reduction in sympathetic nerve activity, but controlled breathing alone without meditation does not alter sympathetic activity in CKD [57].

Studies focusing specifically on self-management among African Americans or older African American adults with chronic kidney disease are particularly limited. Washington et al. [49] conducted a qualitative inquiry that uncovered self-management “helps and hindrances” among older African American and older White individuals undergoing hemodialysis, and found having a social network, following treatment orders, exercise, and religious faith were among the “helps”, while functional limitations, managing co-occurring conditions, and dietary restrictions were “hindrances”. This research has implications for developing culturally relevant self-management interventions for older African American adults with kidney disease through providing clarity regarding the cultural and social contexts that older African American adults bring to the medical encounter. Kahn and colleagues [12] conducted semi structured interviews with a sample that was predominantly African American (79%) and found that four major themes emerged when studying this population regarding self-management: “making sense” of CKD, enlisting support and personal resources, collective action, and monitoring their own illness. Bowling et al. [60] explored factors facilitating or impeding CKD self-management in older adults with a sample that was 60% African American with a mean age of 75.1 years and found that managing complexity of treatment and dealing with co-occurring conditions were central themes that emerged from their participants.

Conceptually, the literature indicates that self-management is complex and limited attention has been given to older African Americans with chronic kidney disease. Embodiment theory proposes that biocultural consequences of social inequities influence individual and societal ways of living. This provides an explanation for how embodied identity is impactful for older African Americans regarding self-management behaviors. These pathways of embodiment are structured by (1) societal arrangements of power, property, contingent patterns of production, consumption, and reproduction; and (2) constraints and possibilities of our biology, as shaped by our evolutionary history, our ecological context, and individual histories, which extend into trajectories of biological and social development [61]. The experience of being both an older adult and African American in the United States informs the conceptualization and execution of self-management behaviors. This is especially salient for the management of chronic kidney disease.

### 1.6. Embodiment Theory

Embodiment theories and approaches are an important tool for anthropological and clinical understandings of the consequences that long-term stress and discrimination have on human bodies. In the broadest sense, embodiment focuses on how bodies are oriented to and shaped by their environments and lived, subjective experiences. It recognizes that humans are simultaneously social beings and biological entities, emphasizing the inseparability of the individual from their daily context [62,63,64,65,66]. Studying the ways people experience social inequalities and suffering through their bodies demonstrates how macrolevel structures, ideologies, and processes can powerfully shape an individual’s—and community’s—exposure to factors that create certain kinds of embodied health outcomes, as well as their responses to sickness, long-term chronic disease, increased financial burdens, and emotional strain [67,68].

In their seminal work discussing the necessary deconstruction of the human body and its orientation as a site of experience and meaning, medical anthropologists Nancy Scheper-Hughes and Margaret Lock [69] explore how the body is simultaneously phenomenologically experienced by the individual, a symbol for social and cultural relationships, and a body politic to be governed and surveilled. They challenge a variety of medical discourse assumptions regarding how and why certain kinds of bodies are socially produced and encoded with meaning. Health in the United States is principally viewed as an achieved status where each individual is expected to “work hard” at being fit and healthy [69], and is expected to have a *moral self-discipline* in avoiding junk food and lethargy. The body and its moral health status are thus the responsibility of the individual, and the clinician is tasked with “fixing” the failures of the diseased, often “non-compliant,” patients.

Yet our medical terminology lacks appropriate terminology to deal with body–society interactions, and many theorists, researchers, and clinicians struggle to view the biological bodies of humans as integrated with the experiences and narratives of illness and suffering. Often, as a result, the illness dimension of human distress (the social relations and experiences of sickness, symptoms, and suffering) become medicalized and individualized, rather than politicized and collectivized. This medicalization then neglects the intersections between the individual and the social bodies by pathologizing social issues embedded in institutional structures, transforming the social into the biological, and perpetuating an individual vs. society opposition [70,71,72,73,74,75,76].

It is no accident that population patterns of health and disease contour the distribution of power, property, and technological resources throughout the world. Generations of access to living wages, adequate insurance coverage, support for child care, sanitation regulations, immunizations, and fresh food combine to promote the distribution of community health and wellness. However, the inverse is true as well: food insecurity, “fast food”-only food deserts, inadequate sanitation, economic and social deprivation and discrimination, physical and sexual abuse, and inadequate preventative healthcare all leave their marks on the human body. Bodies “bear the mark” of their engagement in the world, and social conditions are manifested in physical, chemical, and phenotype changes within the body [77]. Embodiment is this process and concept of how humans biologically incorporate the societal and ecological circumstances in which we live, and it thus contextualizes the responses, attitudes, beliefs, and interpretations of individual patients.

This focus on the larger context of a patient’s health behavior is similarly highlighted by ecosocial theory and models of social determinants of health, yet embodiment is unique in an essential manner. Ecosocial theory explicates the social construction of illness using a multidimensional and multilevel perspective that considers how societal conditions affect health [77]. Ecosocial theory considers the importance that political, social, and economic processes have in shaping disease risk for different groups at individual, neighborhood, regional, and national levels [77]. Older African Americans occupy a unique status within the United States since this group experiences a “double jeopardy” where age and race influence pathways to exposure and susceptibility to various diseases and comorbidities. A particularly salient example of how race can influence pathways to disease development is the case of hypertension, the second leading cause of chronic kidney disease in African Americans. Enduring racial discrimination leads to residential and occupational segregation and economic inequity among African Americans, and is specifically associated with nutritionally poor or low-quality food opportunities in those communities [68,77,78]. Thus, the risk of hypertension is compounded in a multitude of ways: high-fat, high-salt, and low-vegetable diets directly increase risk; poor accessibility to outdoor community green spaces for exercise or neighborhood activities; hourly wage or low-salary jobs provide little to no healthcare insurance protections; economic deprivation increases the likelihood of pre-term birth with insufficient kidney development, leading to an increased likelihood of chronic salt retention; and, consequently, a combination of a biological predisposition, such as chronic salt retention, and limited healthy food options on a daily basis provide few possibilities for individuals to avoid hypertension development or disease progression. It is a cycle where social inequalities shape the biology of racialized groups, and then embodied inequalities perpetuate a position of imbalanced health and wellness [79].

Many social science health researchers, sociologists, and social epidemiologists also laud the utility of social determinants of health approaches. This approach acknowledges that the factors which determine health and health outcomes are complex, imbedded in larger social structures, and not likely to be adequately addressed within the healthcare delivery system itself. When discussing cross-sectional health variation, social determinants usually provide more explanatory power than differences in medical care [80]. These social determinants of health include socioeconomic status; education level; racial or ethnic discrimination; availability of safe housing; residential segregation; environmental pollution levels; nutrition; occupation; public safety; social support; transportation options; exposure to crime and/or violence; language or literacy; and cultural norms and values [81]. These types of social conditions can be considered the “fundamental causes” of disease once these multilevel social mechanisms are examined and individual risk factors are contextualized [82]. The tendency of medical systems to focus on the proximate causes of physiological disease neglects the multilayered and variable processes through which social factors affect health [82,83].

All three approaches to the ultimate causes of disease—embodiment, ecosocial, and social determinants of health perspectives—move beyond reporting the mere distribution of disease, and seek to understand how institutional and interpersonal factors influence susceptibility, exposure, and persistence. Yet what makes embodiment theory unique, and, thus, why we believe it is a vital addition to the concept of self-management, is its direct promotion of the need to treat the patient, not the disease. While acknowledging the fundamental importance of macrolevel statistical analyses and structural violence issues, the work of embodiment theory is to incorporate those systemic understandings into the direct interaction with—and treatment of—the patient. From the perspective of embodiment, the awareness of structural forces, societal limitations, and social origins of disease within a healthcare setting is not enough; it requires the incorporation of these understandings into the individual-level treatment plans with the acknowledgement that population-level patterns and statistics are distant abstractions of patients’ realities. Only when there is a combined understanding of the unique individual patient, the social structure and cultural limitations that they must navigate within, and the medical regimens needed to achieve optimal chronic disease management, can healthcare be truly patient-centered and achieve the highest level of management capability for the greatest number of patients.

## 2. Discussion

Connecting embodiment theory to chronic kidney disease self-management is a novel approach that provides a rationale for including the experiences patients and families bring to their own care management and using these experiences to understand how individuals define and shape self-management behaviors. This perspective acknowledges that patients do have responsibility for their care, but requires that the healthcare system reflexively evaluate established, but limited, definitions of self-management that do not consider the legitimacy of patient context in making decisions regarding self-management. It is presumed that high-quality management of chronic illnesses can only be achieved if patients “take responsibility” and are invested in “taking charge” of their own health. This concept is reflected as self-management and is said to provide increased autonomy for the patient, improve quality of life and self-efficacy, and reduce the burden of specialized centers [84]. However, based on these underlying morals of responsibility and self-motivation, when patients are unable to “comply” with this current model of self-management, the reverse is then presumed about them: the patient must not be “trying hard enough,” is unable to adapt their lifestyle, cannot maintain a positive mind, cannot achieve personal competence, or will not take initiative through self-motivation. Not only does this ignore the context of the patient’s embodied lived reality and illness experience, it also relieves the healthcare system of any liability: if a patient is unwilling to comply with the recommendations of a treatment plan, then the healthcare system has “done all it can,” as opposed to a patient being unable (but willing) to adhere to the recommendations because of social and structural barriers.

Considering how racial identity, ageism, and the pathways to developing chronic kidney disease among older African Americans are distinct, patients’ self-management behaviors are also uniquely situated: the ways in which this illness is managed among individuals is inevitably influenced by a combination of social, political, and economic processes that limit self-management avenues. A patient’s decisions about managing their care have to be made in the context of their current lives and lived experiences, which may be markedly different than those providing healthcare services. It is then clearly important for healthcare workers and providers to understand that social factors are embodied and enacted within the context of self-management decision-making for older African American adults. This shift in defining self-management care will move towards a more patient-centered paradigm. As a construct and process, embodiment is a critical tool for understanding how and why historically contingent, spatial, temporal, and multilevel processes become embodied and generate population patterns of health, disease, and wellbeing. By systematically conceptualizing self-management in relation to embodiment and utilizing a holistic paradigm of patient care, it will be possible to not only inform clinical practice and community-based strategies aiming to improve disease-specific and overall health outcomes for older African Americans, but to enhance knowledge relevant to attaining more socially equitable healthcare.

Older African American adults stand to benefit from an increasingly patient-centered approach that shifts the focus of healthcare from being provider-led to patient-led. Despite beginning efforts to examine the experiences of African Americans in care systems and understand the impacts of structural racism and perceived discrimination, these factors continue to impact how African Americans are cared for within the healthcare system. Practices such as presuming poor health status and outcomes as “normal” for African Americans, unethical experimentation on African Americans, racial bias in clinical decision-making, and the assignment of Black and other ethnic minorities to inferior tiers of the health delivery system have been mentioned in prior research as factors that impact how African Americans view and are viewed by the healthcare system [85]. More importantly, these factors have influenced how African Americans have adapted self-management behaviors responding to a healthcare system that has not provided adequate care for African Americans.

Chronic kidney disease is an illness that requires significant self-management ability, but also utilizes a great deal of interdisciplinary care. This style of treatment, which is a hallmark of chronic kidney disease care, is an appropriate fit for person-centered care in which patients and physicians take responsibility for disease management. Person-centered care perspectives are now increasingly accepted in the medical field; however, these perspectives lack theoretical underpinnings that explain why social factors shape how and why patients make certain decisions about managing their own care. Embodiment theory provides a lens through which to examine why certain self-management decisions are practical within the paradigms that patients bring to the healthcare encounter. For researchers, collaborations across disciplines are key to discovering new ways to understand how patient context can influence chronic kidney disease self-management. Among practitioners these perspectives can spur innovative approaches to self-management through utilizing the entirety of the interdisciplinary team. One such example of utilizing interdisciplinary teamwork in chronic kidney disease self-management care is the case of social work.

Social work is well positioned to have an impact in chronic kidney disease self-management care, easing the burden on physicians and nurses while focusing on specific facets of the social context that influences self-management behaviors. Currently, social workers are mandated to be involved in chronic kidney disease care only once a patient progresses to the final stages of chronic kidney disease and requires dialysis. End Stage Renal Disease (ESRD or Stage 5 chronic kidney disease) is the only disease category with a public policy inclusion (Medicare) for master’s level social workers on health teams [86]. Self-management skills, such as developing motivational plans, goals, and concentration skills, are features of this concept that go hand-in-hand with the mission of the social work profession to empower especially at-risk, marginalized populations such as older African American adults

## 3. Conclusions

Finally, anticipating the projected influx of renal patients is key to managing healthcare expenditures. For U.S. adults aged 50 to 64, and 65 years or older with no CKD at baseline, the residual lifetime incidences of CKD are 52%, and 42%, respectively [87]. It is critically important for the healthcare system to be able to effectively treat patients who have been alienated from the healthcare system and have adopted a specific set of self-management skills out of necessity, but are now forced to interact with medical systems who label patients as noncompliant if they do not strictly adhere to the medical model of self-management care. Older African Americans are living with the historical burdens of unequal treatment coupled with the burden exacted by current political rhetoric and increased exposure to racially charged incidents. This environment reinforces historical traumatization and amplifies mistrust in social institutions such as the healthcare system.

Living with chronic kidney disease places older African Americans in a vulnerable position, where healthcare self-management behaviors borne out of necessity are subject to great scrutiny in healthcare settings, and are often met with a lack of empathy and understanding. Utilizing embodiment theory to understand the patient context and to provide a rationale for activating true multidisciplinary, person-centered care is integral to meeting the needs of older African Americans entering the renal care system.
